# Supplementary figures and images for: DNA polymerase swapping in Caudoviricetes bacteriophages
Source: Virol J. 2024 Aug 26;21:200. doi: 10.1186/s12985-024-02482-z (PMC11348598; doi:10.1186/s12985-024-02482-z)

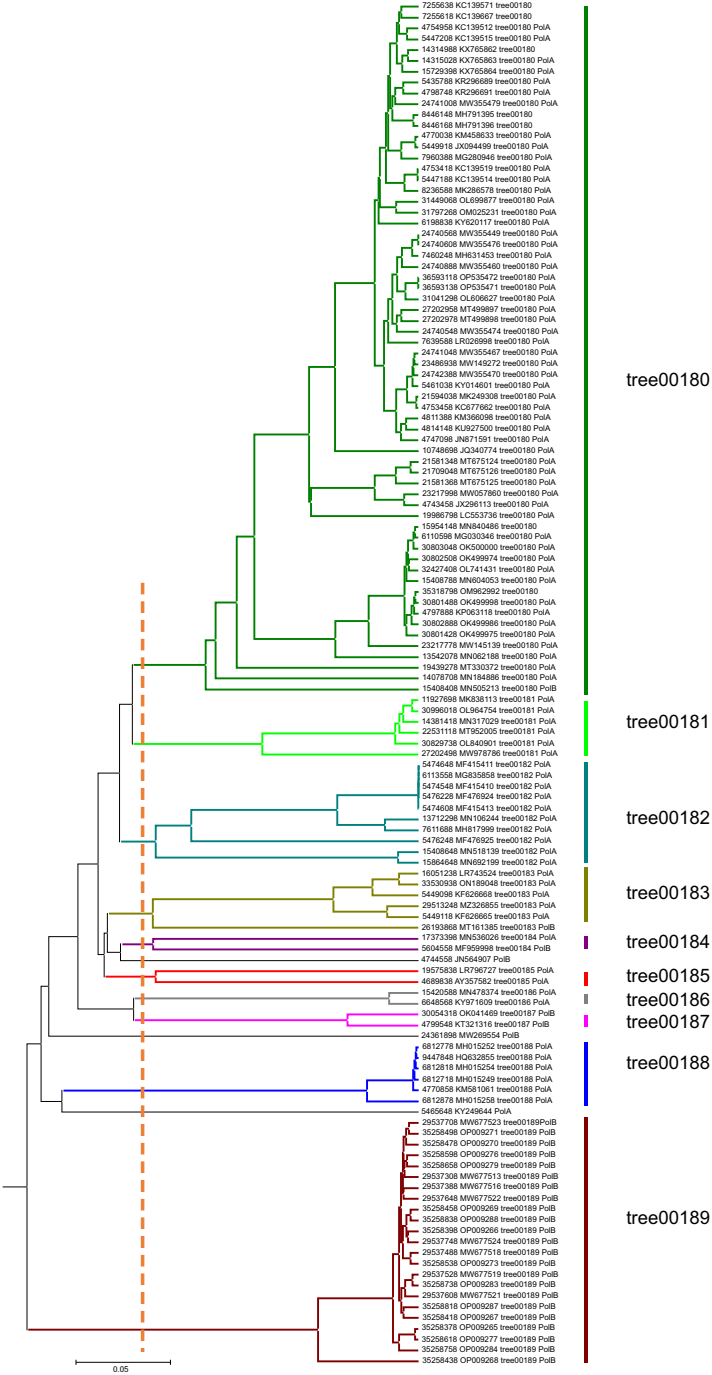

PolA

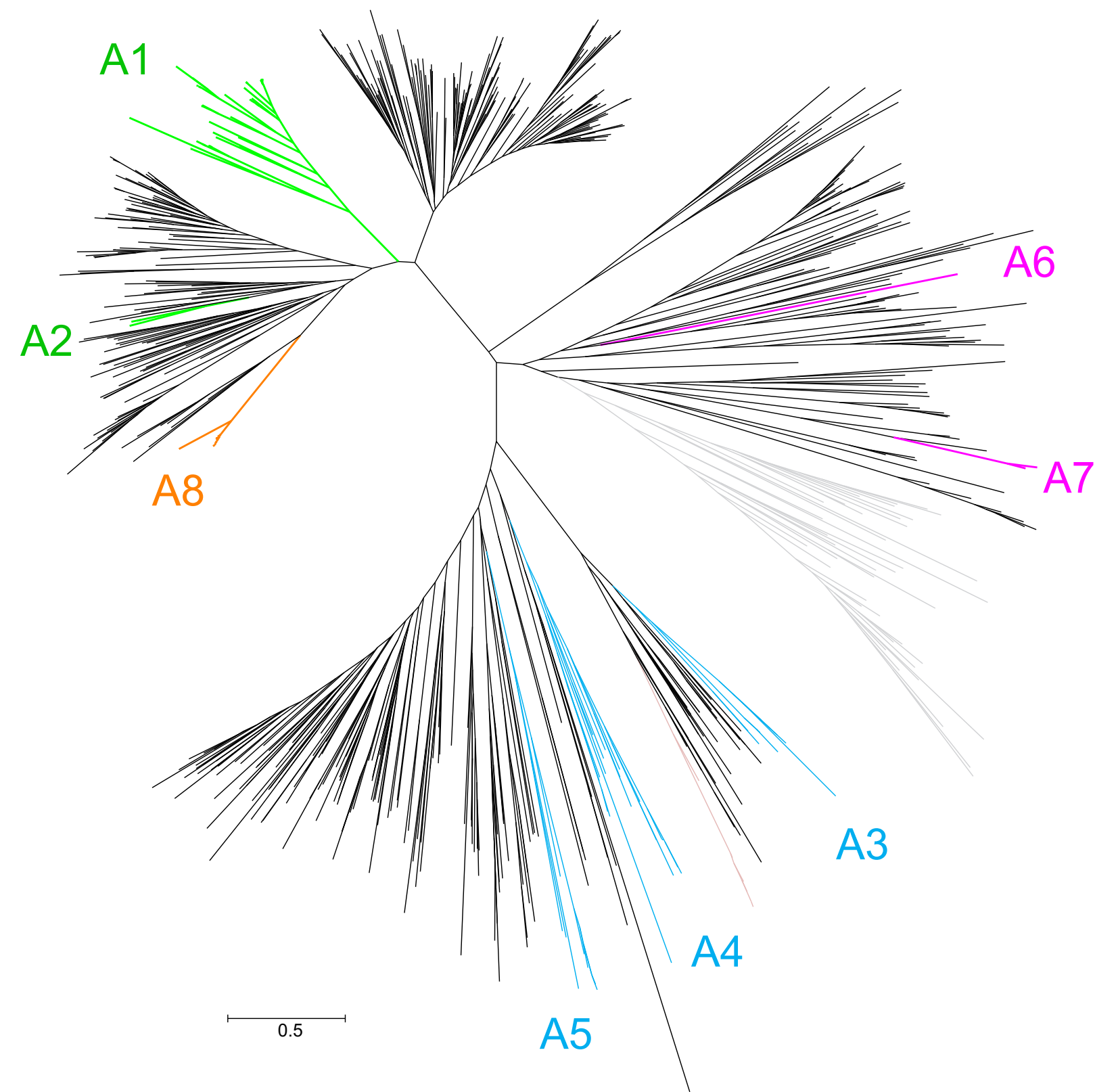

PolB

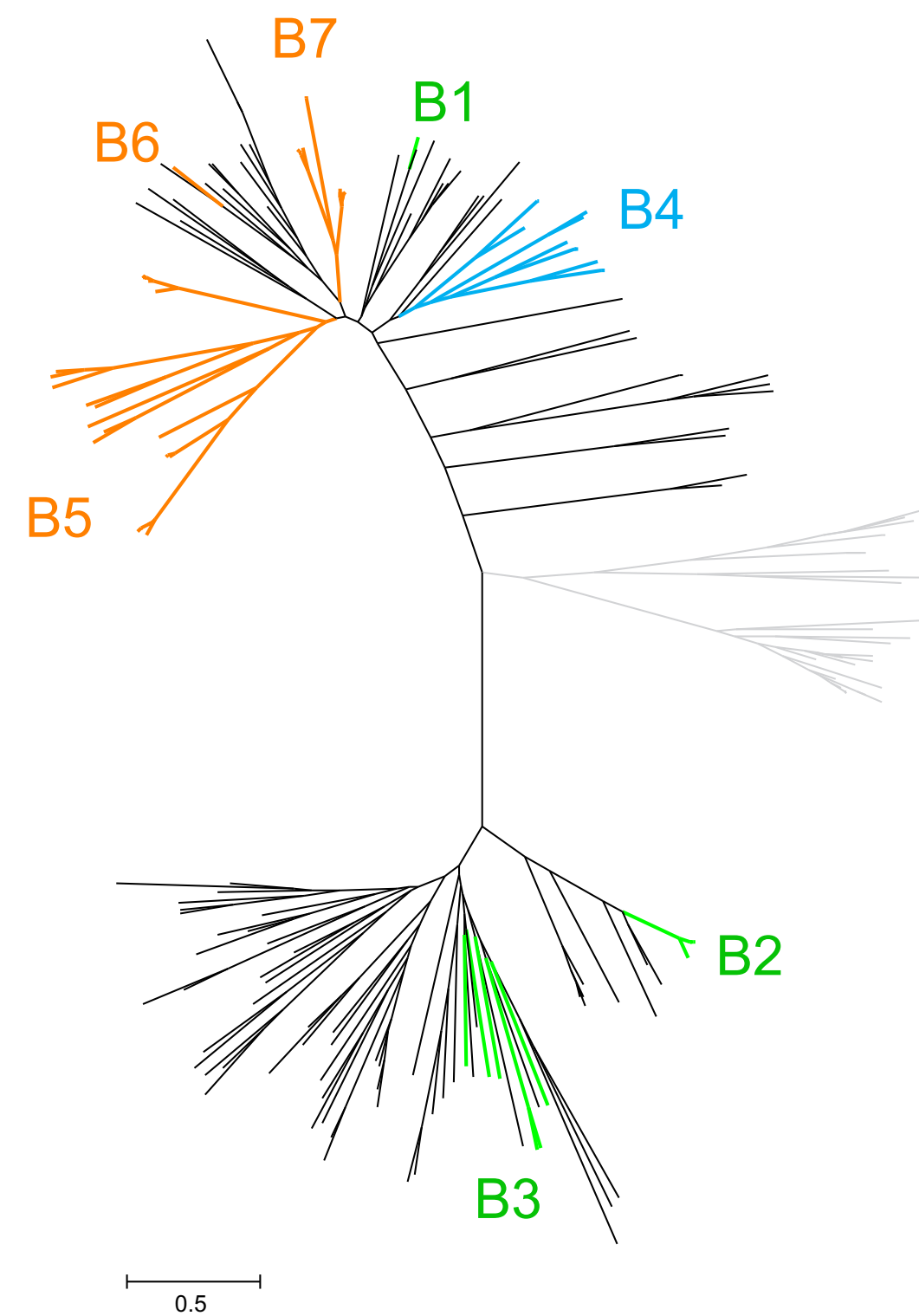

PolC

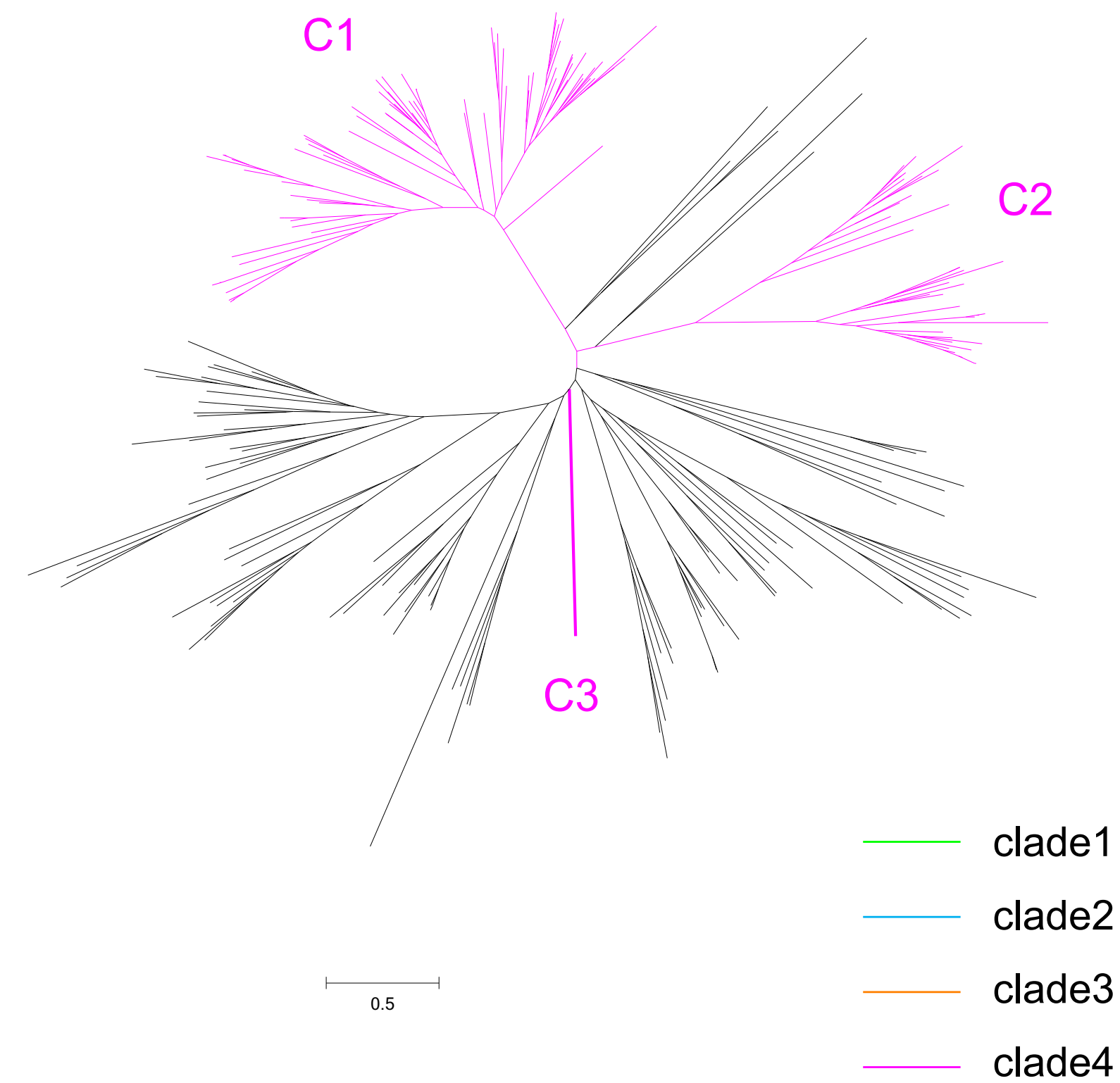

- clade1
- clade2
- clade3
- clade4

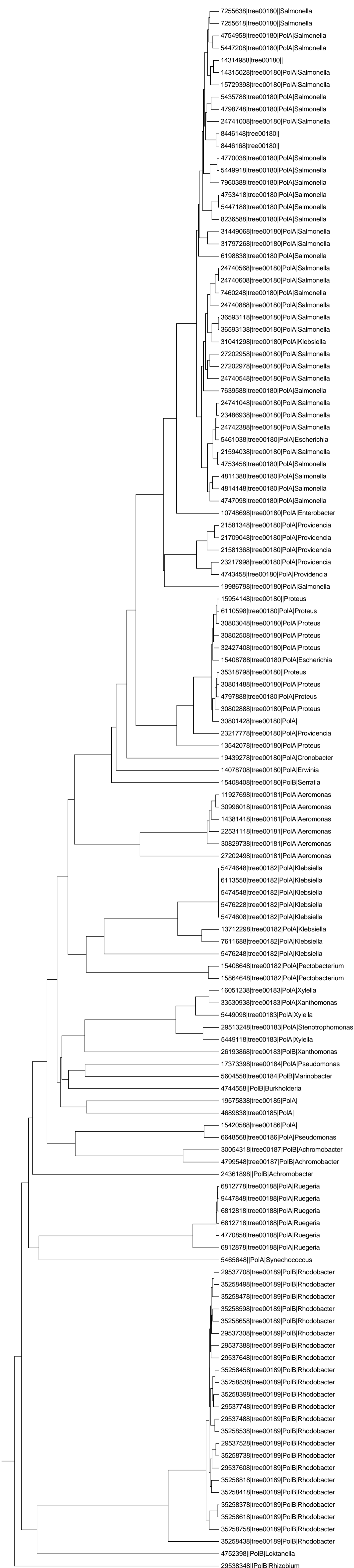

0.050

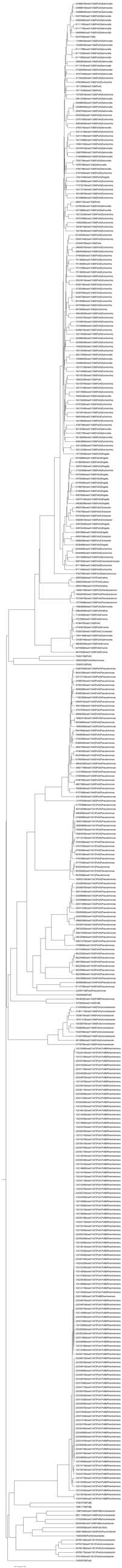

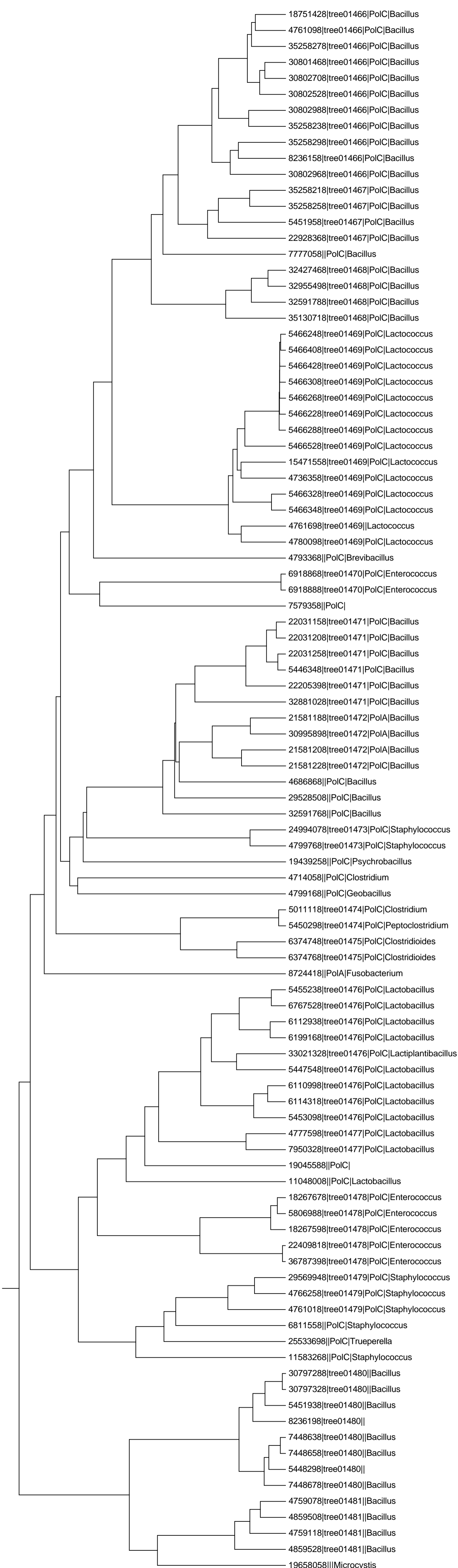

0.10

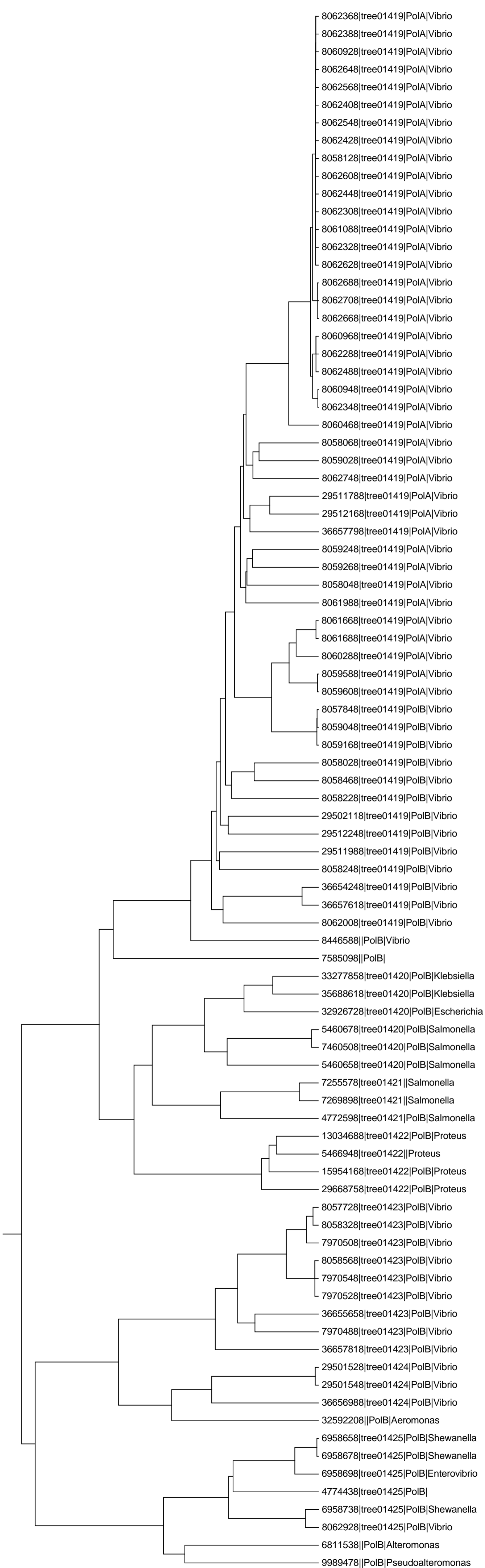

0.050

LR796859 LR796859 1..26372

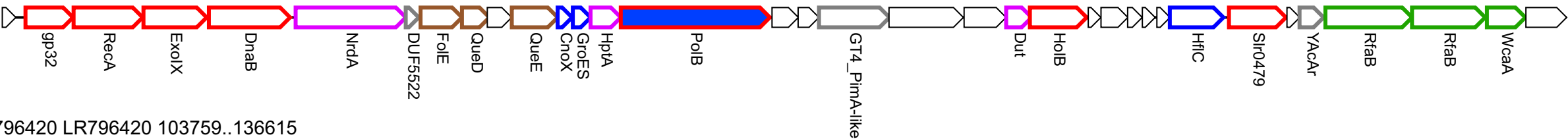

LR796420 LR796420 103759..136615

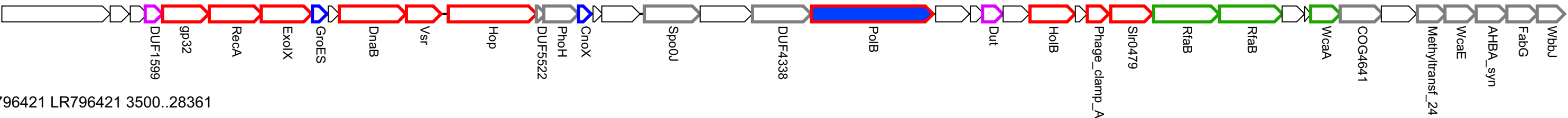

LR796421 LR796421 3500..28361

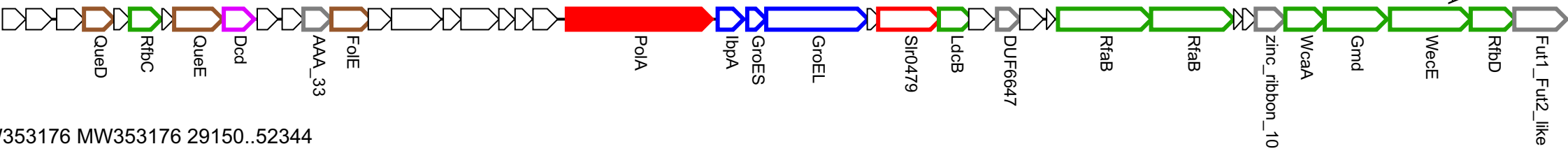

MW353176 MW353176 29150..52344

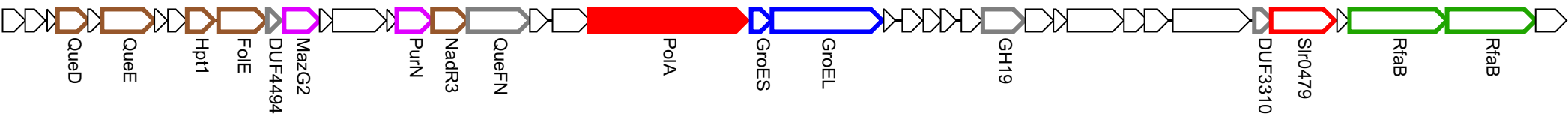

LR796188 LR796188 136850..113176

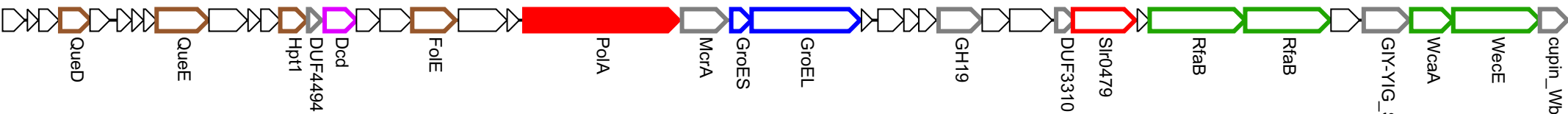

LR796345 LR796345 51203..78226

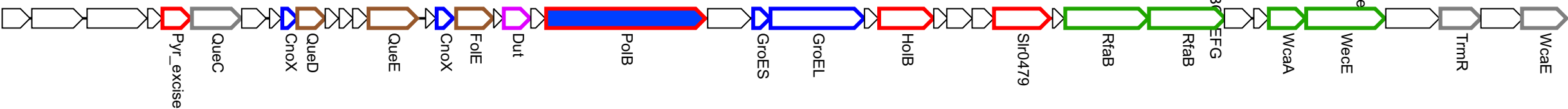

MK892766 MK892766 36299..7410

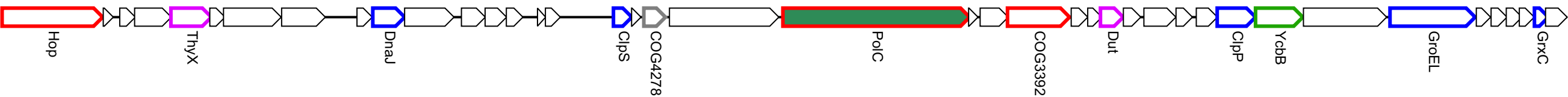

MK892784 MK892784 128296..159733

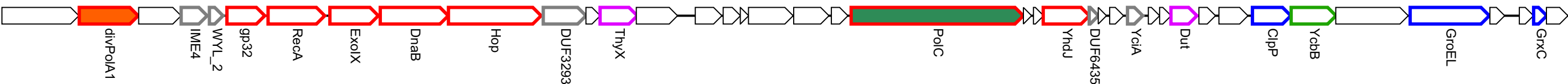

10000 nt

Supplement: Supplementary file 3 — Supplementary Material 3 [file 12985_2024_2482_MOESM3_ESM.pdf]
